# Supplementary material for: A network dynamical simulation model for the study of antibiotic resistance in microbial communities
Source: Gut Microbes. 2026 Sep 19;18(1):2734703. doi: 10.1080/19490976.2026.2734703 (PMC13596919; doi:10.1080/19490976.2026.2734703)
Supplement: Supplementary file 1 [file KGMI_A_2734703_SM9053.zip › supplementary_file_1/Figures/er_delta_n_components_n300.pdf]

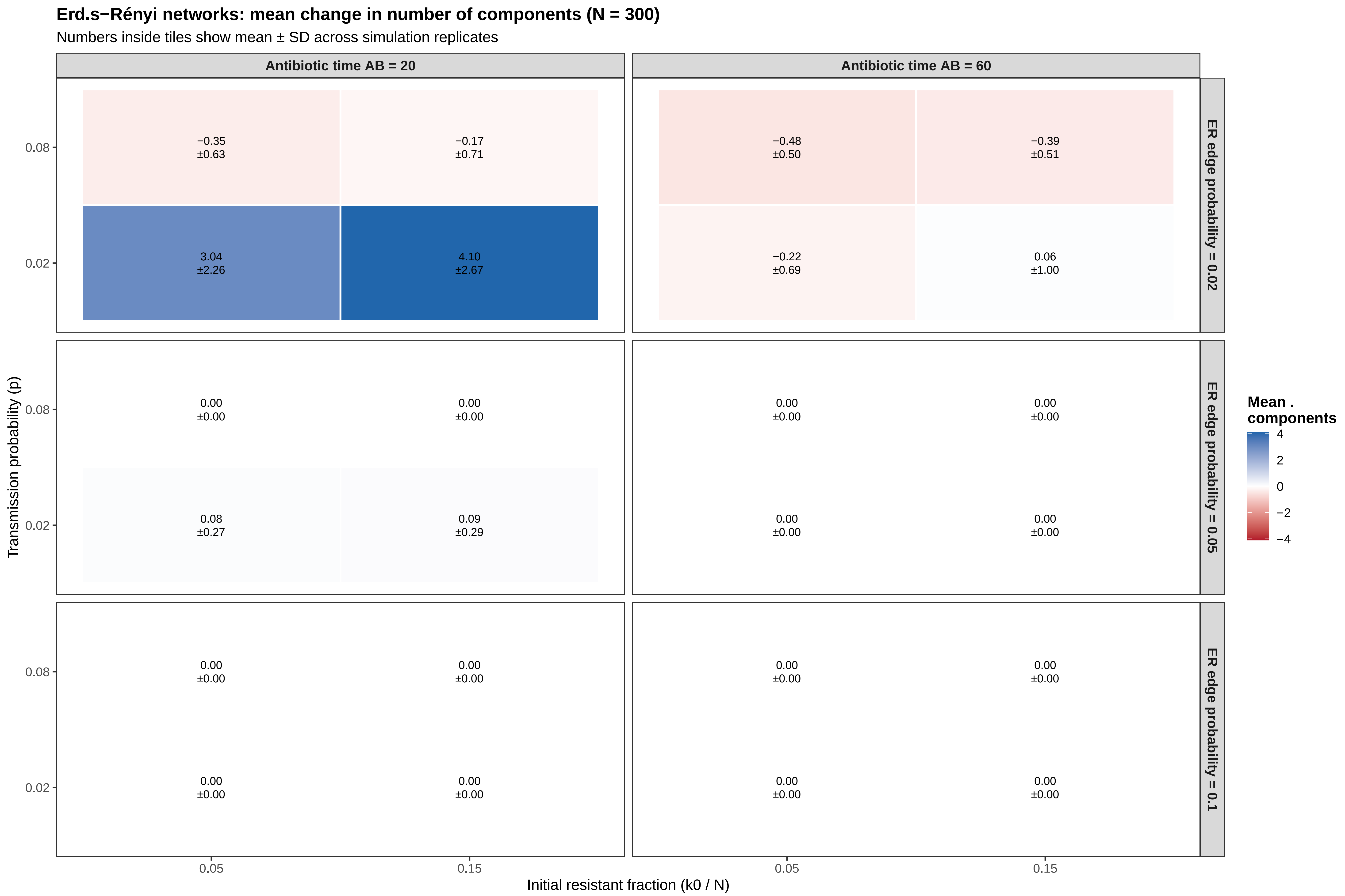

Erd.s−Rényi networks: mean change in number of components (N = 300)
Numbers inside tiles show mean ± SD across simulation replicates
Antibiotic time AB = 20
Antibiotic time AB = 60
ER edge probability = 0.02
ER edge probability = 0.05
ER edge probability = 0.1
−0.35 ±0.63
−0.17 ±0.71
3.04 ±2.26
4.10 ±2.67
−0.48 ±0.50
−0.39 ±0.51
−0.22 ±0.69
0.06 ±1.00
0.00 ±0.00
0.08 ±0.27
0.09 ±0.29
Transmission probability (p)
0.08
0.02
Initial resistant fraction (k0 / N)
0.05
0.15
Mean . components
4
2
0
−2
−4
